# Behavioural Determinants of Intestinal Nematode Infection Risk Among Children in a Post-Mass-Drug-Administration Setting in Sri Lanka: A Survey of Caregiver Knowledge, Attitudes, and Practices

**DOI:** 10.3390/tropicalmed11070191

**Published:** 2026-07-09

**Authors:** Nalini Jayakody, Catherine A. Gordon, Anjana Silva, Nuwan Wickramasinghe, Susiji Wickramasinghe, Natasha Collinson, Asela Wijayasekara, Chanaka Karunarathne, Harshi Weerakoon, Manjula Weerasinghe, Nilanthi de Silva, Kosala Weerakoon

**Affiliations:** 1 Department of Parasitology, Faculty of Medicine, Wayamba University of Sri Lanka, Kuliyapitiya 60200, Sri Lanka; asela@wyb.ac.lk; 2 Department of Parasitology, Faculty of Medicine and Allied Sciences, Rajarata University of Sri Lanka, Saliyapura 60200, Sri Lanka; anjana@med.rjt.ac.lk; 3 QIMR Berghofer Medical Research Institute, Infection and Inflammation Program, Applied Tropical and Molecular Parasitology Laboratory, Brisbane, QLD 4006, Australia; 4 Faculty of Medicine, The University of Queensland, Brisbane, QLD 4067, Australia; 5 Center for Tropical Health and Emerging Diseases, QIMR Berghofer Medical Research Institute, Brisbane, QLD 4006, Australia; 6 Department of Community Medicine, Faculty of Medicine and Allied Sciences, Rajarata University of Sri Lanka, Saliyapura 50300, Sri Lanka; nuwick@med.rjt.ac.lk (N.W.); manjugaya@yahoo.com (M.W.); 7 Department of Parasitology, Faculty of Medicine, University of Peradeniya, Kandy 20400, Sri Lanka; susiji.wickramasinghe@med.pdn.ac.lk; 8 Department of Physiology, Faculty of Medicine, Wayamba University of Sri Lanka, Kuliyapitiya 60200, Sri Lanka; chanaka85@wyb.ac.lk; 9 Department of Biochemistry, Faculty of Medicine and Allied Sciences, Rajarata University of Sri Lanka, Saliyapura 50300, Sri Lanka; harshitw@med.rjt.ac.lk; 10 Department of Parasitology, Faculty of Medicine, University of Kelaniya, Ragama 11010, Sri Lanka; nrdesilva@kln.ac.lk

**Keywords:** caregiver behaviour, human intestinal nematode infections, knowledge, attitudes and practices (KAP), mass drug administration, post-elimination surveillance, schoolchildren, soil-transmitted helminths

## Abstract

Human intestinal nematode infections (HINIs) remain a public health concern despite reduced prevalence following mass drug administration (MDA) in low- and middle-income countries. Children continue to bear a substantial burden of infection, and caregiver knowledge and practices may influence their risk of acquiring infection. Sustaining control during the transition of prevention programmes to surveillance-based strategies requires an understanding of behavioural determinants of transmission. Therefore, we assessed caregiver knowledge, attitudes, and practices (KAP) and their associations with sociodemographic characteristics and child infection status. A community-based cross-sectional study was conducted among 945 caregivers of primary school children in Anuradhapura, a low-prevalence setting where school-based MDA ceased in 2019, to assess caregiver KAP and child HINI status. An interviewer-administered questionnaire assessed KAP, sociodemographic, water, sanitation, hygiene (WASH), and behavioural factors. Participants were predominantly mothers (89.5%). Good knowledge was observed in 61.7%, positive attitudes in 95.6% and good practices in 99.2%. Knowledge gaps persisted regarding transmission and complications. Higher caregiver knowledge (OR = 0.52; 95% CI: 0.36–0.72) and regular deworming (OR = 0.58, 95% CI: 0.40–0.83) were associated with lower odds of infection, whereas attitudes and practices were not independently associated. Caregiver knowledge remains a key determinant of infection risk. Gaps in knowledge, misconceptions, and practices may sustain transmission even in low-prevalence settings. Strengthening health education, school-based hygiene promotion, and community engagement remains essential to reinforce preventive behaviours and support long-term control as countries transition from routine MDA to elimination-oriented strategies.

## 1. Introduction

Human intestinal nematode infections (HINIs) continue to pose a significant global health challenge, particularly in low- and middle-income countries [1]. A subset of these infections is caused by soil-transmitted helminths (STH), including *Ascaris lumbricoides*, *Trichuris trichiura*, *Necator americanus*, *Ancylostoma* spp., and *Strongyloides stercoralis* [1,2]. *Enterobius vermicularis*, although not classified as an STH, is also globally prevalent. Together, these HINIs affect an estimated 1.5 billion people worldwide [1]. Transmission occurs via the faecal-oral route or skin penetration, depending on the species [1]. Inadequate water, sanitation, and hygiene (WASH), including open defecation and unsafe water, facilitate environmental contamination and sustained transmission [2]. Although many infections are often asymptomatic or present with mild non-specific symptoms like nausea, weight loss, pruritus ani, and fatigue, they can impair daily functioning and school performance [2]. Chronic or high-intensity infections can lead to malnutrition, anaemia, stunting, cognitive impairment, intestinal obstruction, disproportionately affecting children, pregnant women, immunocompromised people and individuals in impoverished settings [3].

Mass drug administration (MDA) has been the cornerstone of global control strategies for HINIs, substantially reducing infection intensity and related morbidity in many endemic regions [4]. However, in some settings, reinfection remains common due to continued exposure to contaminated environments and the rapid re-establishment of transmission cycles, which are influenced by gaps in knowledge, attitudes, and practices (KAP), alongside structural constraints such as poverty and inadequate WASH [5,6,7,8]. KAP is a framework for assessing what individuals know about a health issue, their perceptions and beliefs, and their corresponding practices. It is widely applied in public health research to identify gaps in knowledge and determinants influencing disease prevention and control [9]. Limited knowledge impairs disease recognition and understanding of transmission routes, while negative attitudes, characterised by low perceived susceptibility and severity, diminish motivation for protective behaviours [6,7,8]. Poor hygienic practices further increase exposure risk [6,7,8]. Evidence from endemic regions further indicates that awareness alone is insufficient, with fewer than 20% of individuals in some settings consistently practising adequate preventive measures [6,7].

This challenge is particularly relevant in settings transitioning from morbidity control to elimination, where sustained behavioural change and community awareness are essential to prevent continued transmission [6,7]. The magnitude and nature of these behavioural gaps may differ across communities with varying levels of endemicity, highlighting the need for context-specific evidence to guide interventions [10,11]. The World Health Organization (WHO) neglected tropical diseases roadmap 2021–2030 emphasises integrated strategies combining MDA with WASH, and effective health education [11,12].

In Sri Lanka, HINIs have been documented since the late nineteenth century [13]. Over subsequent decades, sustained control efforts, including school-based MDA integrated with antifilarial campaign and national WASH improvements, have been implemented nationwide [14,15]. These efforts have reduced STH prevalence from 98% to below 1% by 2017, placing the country close to the elimination of the disease as a public health problem [13,16]. In 2019, routine MDA was discontinued in low-prevalence districts, including Anuradhapura, Kurunegala, and Batticaloa [16,17]. While this transition represents a major public health milestone, it also increases the risk of resurgence if preventive behaviours are not maintained, as demonstrated by previous increases in infection following interruptions to control programmes in plantation communities during the 1990s [18]. Anuradhapura district, now reporting very low prevalence (0.21%), exemplifies this critical transition phase and was selected as the setting for the present study.

Health education interventions can improve knowledge, preventive practices, and participation in control programmes [7,19]. Caregivers are central to shaping children’s hygiene, food safety, and healthcare-seeking behaviours, making their KAP critical determinants of infection risk at the household level. Children remain among the most vulnerable groups for HINI-associated morbidity.

Despite substantial progress, evidence on KAP and community response in low-prevalence, post-MDA settings remains limited. Understanding these behavioural determinants is essential to sustain gains and prevent continuing transmission, particularly as countries transition towards surveillance-based elimination strategies [11].

Therefore, this study aimed to assess the KAP related to HINIs among caregivers of primary schoolchildren (PSC) in the Anuradhapura district, a sentinel site for national deworming surveillance. As part of a broader community-based survey of intestinal parasitic infections among PSC and their households [20], this analysis focuses on the behavioural component, while epidemiological and diagnostic findings are reported separately. The study further examined variations in KAP according to selected sociodemographic characteristics and the infection status of participating children.

## 2. Materials and Methods

### 2.1. Study Design

This was a community-based cross-sectional study conducted among caregivers of PSC in a low-prevalence setting to assess caregiver KAP related to HINIs. Data were collected from January to November 2023 through a structured questionnaire. The study was reported in accordance with the STROBE guidelines for cross-sectional studies.

### 2.2. Study Location

**Figure 1:**
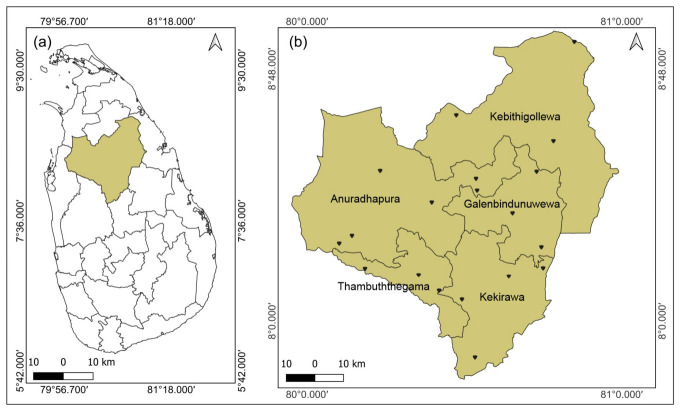
Location map of the study area: (**a**) Sri Lanka with Anuradhapura district demarcations. Lines indicate district margins. (**b**) Anuradhapura district with data collector points (schools). Lines indicate the educational zone margins. Maps were drawn in the Quantum Geographic Information System (QGIS), and the base layer was taken from DIVA-GIS (https://diva-gis.org/).

Sri Lanka is a lower-middle-income island nation in South Asia. It is divided into nine first-level administrative divisions (provinces) and 25 second-level divisions (districts). Anuradhapura (Figure 1a) is one of two districts in the North Central Province, with an elevation of 81 m above sea level [21]. The district is located in the dry zone, with an average annual temperature of 28.1 °C, and annual precipitation of around 1287 mm spread across 160 rainy days [21]. Covering approximately 7179 km^2^, the district had an estimated population of over 860,000 in 2021, corresponding to a population density of 129 people per km^2^ [22]. Around 94% of the population lives in rural areas [22]. The population is predominantly Sinhalese (91%), followed by Sri Lankan Moor and Tamil communities [22]. Agriculture and animal husbandry constitute major economic activities [23]. Drinking water is obtained from piped water supply schemes operated by the National Water Supply and Drainage Board (NWSDB), protected dug wells, tube wells, and rainwater harvesting systems [23]. In areas affected by chronic kidney disease of uncertain aetiology (CKDu), community-level reverse osmosis (RO) treatment plants have been introduced to improve access to safer drinking water [23]. Sanitation coverage is high and predominantly based on on-site sanitation systems, mainly pour-flush latrines connected to pits or septic tanks, with improved sanitation access exceeding 90% [23]. Despite relatively good WASH infrastructure coverage, gaps remain with limited WASH facilities in some rural schools [23,24,25,26]. The district has five educational zones (Figure 1b), comprising 817 schools classified into types 1AB, 1C, 2, and 3 categories [27]. Type 1AB and 1C schools provide education up to the Advanced Level; Type 2 schools provide education up to the Ordinary Level; and Type 3 schools provide primary education only.

### 2.3. Study Population and Sample Size

This study was part of a larger school-based, cross-sectional survey conducted in the Anuradhapura district of Sri Lanka to assess the prevalence of HINIs among children in Grades 1–5 (ages 5–11 years) attending state schools [20]. A total of 19 state schools were selected using multistage stratified cluster sampling. Educational zones were used as strata, and schools within each zone were selected by simple random sampling using computer-generated random numbers, as described previously [20]. The number of schools included (*n* = 19) was determined as part of the sampling framework developed to achieve the required sample size for the prevalence survey while ensuring representation from all five educational zones of the district. The present component focused on assessing the KAP of parents or caregivers of these children, as well as child-related practices reported by the caregivers.

In this study, a caregiver was defined as any adult responsible for the day-to-day care and supervision of the primary school child, including parents, grandparents, or other household members who regularly care for the child. All parents or caregivers of the selected students were eligible for inclusion, and no exclusion criteria were applied.

The sample size was calculated for prevalence assessment using the standard single-proportion formula [16,28]. An expected prevalence of 5% was considered using a 95% confidence interval (95% CI) and a precision of 2% (Z = 1.96, *p* = 0.05, d = 0.02). To account for clustering and potential sampling variability, a design effect of 1.5 was applied, which adjusts for the increased variance expected in cluster-based or multi-stage sampling compared to simple random sampling, resulting in a sample size of 688, and was further increased by 30% to account for anticipated non-compliance.

### 2.4. Data and Sample Collection

Data were collected using an interviewer-administered questionnaire comprising three domains: socio-demographic information, KAP data and deworming information. The questionnaire was developed based on an extensive review of relevant literature on KAP surveys related to intestinal parasitic infections. Items were adapted from previously published studies, and additional questions were generated to reflect the local epidemiological and socio-cultural context of the study population. The initial draft was prepared in English. The questionnaire was subsequently translated into Sinhala, and cultural and contextual adaptation was undertaken to ensure suitability and comprehensibility for the local study population. Face and content validation of the questionnaire were carried out through review by a panel of experts in parasitology, public health, and community medicine. In addition, the translated questionnaire was evaluated by non-expert individuals representing a population similar to the target study participants to assess clarity, comprehensibility, and acceptability. The questionnaire was pretested in a similar community setting prior to data collection. Based on feedback obtained during expert review and pretesting, minor modifications were made to improve wording, clarity, and contextual appropriateness before final administration.

The interviews were conducted in the Sinhala language, as all the participants were Sinhalese. The interview protocol served as the primary guide to reduce interviewer bias, with some flexibility in following up on interviewees’ statements using additional probes and questions. Participants in the study were not paid monetarily, but each participating child was given a small gift as a gesture of gratitude for their participation.

As part of the broader survey, stool samples were collected from participating children and examined for HINs. Infection status was included in the present analysis only to explore potential associations between caregiver KAP and child infection status. The behavioural (KAP) and parasitological findings are reported separately to allow focused interpretation of behavioural determinants and epidemiological outcomes.

### 2.5. Data Analysis

Knowledge was assessed using eight primary questions comprising 33 individual response items. Five questions had “Yes/No” response options, while 3 included “Yes/No/Not sure.” Each correct response was given a score of 1, and incorrect or “don’t know/not sure” responses were scored 0, resulting in a total possible score of 0–33 [7]. Attitudes were assessed using 21 statements on a four-point Likert scale with response options of “Agree,” “Neutral,” “Disagree,” and “Don’t know.” For positively framed items, “Agree” was scored as 2, “Neutral” as 1, and both “Disagree” and “Don’t know” as 0, yielding a maximum score of 42. Reverse scoring was applied to negatively framed items [7]. Practices were evaluated using two sets of questions. First, six questions assessed handwashing habits on a five-point Likert scale: “Always with soap and water” (4), “Sometimes with soap and water” (3), “Always with water only” (2), “Sometimes with water only” (1), and “Never” (0). Second, five additional practice questions assessed hygiene behaviours using a three-point scale (“Usually” = 2, “Sometimes” = 1, “Never” = 0), with reverse scoring applied to negatively framed items [7]. The total possible score ranged from 0 to 34.

KAP scores were standardised to percentages and categorised as “good” or “poor”. A priori cut-off values were defined, with scores ≥ 50% classified as good and scores < 50% classified as poor across all three domains: knowledge, attitudes, and practices. Findings from a parallel parasitological evaluation of HINIs conducted among the same cohort of participating schoolchildren were incorporated into the KAP analyses to examine behavioural associations with infection status [20]. Data were entered into a Microsoft Excel database, checked for accuracy, and imported into the Statistical Package for the Social Sciences (SPSS, version 27.0 IBM Corp., Armonk, NY, USA) for statistical analysis. Forest plot was generated using R (version R v4.3.1; R Foundation for Statistical Computing, Vienna, Austria), and the alluvial diagram was created using SankeyMATIC (https://sankeymatic.com). Descriptive statistics, including frequencies, percentages, means, and standard deviations (SD), were used to summarise socio-demographic characteristics and KAP scores. Associations between categorical variables were initially assessed using the Chi-square (*χ*^2^) test. Binary logistic regression analysis was performed to estimate odds ratios (ORs) with 95% CI for factors associated with the presence of HINIs. Independent *t*-tests or one-way analysis of variance (ANOVA) were used to compare mean scores between groups, as appropriate. A *p*-value of <0.05 was considered statistically significant.

Associations between caregiver knowledge, attitudes, and practices and child infection status were analysed using χ^2^ tests and logistic regression models. Infection data were included solely to explore behavioural correlates, while the detailed parasitological findings are reported separately [20].

## 3. Results

A total of 945 parents/caregivers were recruited for participation. All approached caregivers provided written informed consent and completed the questionnaire, yielding a participation rate of 100% with no refusals.

### 3.1. Socio-Demographic and Household Characteristics

The mean age of the participants was 37.1 years (SD: 7.7). The majority were females (*n* = 910, 96.3%). Most participants were mothers of the enrolled children (*n* = 847, 89.5%), followed by grandmothers (*n* = 48, 5.1%), fathers (*n* = 35, 3.7%), sisters (*n* = 8, 0.8%), and aunts (*n* = 7, 0.7%). Nearly all participants were Sinhalese (*n* = 943, 99.8%), with one Muslim (0.1%) and one of Philippine ethnicity (0.1%). Most had completed education up to the senior secondary level (*n* = 610, 64.6%). Two-thirds were not in paid employment and were full-time caregivers (*n* = 639, 67.6%). Among those employed, farming was the most common occupation (*n* = 173, 56.5%). The majority of households used community filtration systems as their primary drinking water source (*n* = 784, 83.0%), followed by dug wells (*n* = 72, 7.6%), with smaller proportions using piped purified water (government supply), bottled water, or rainwater harvesting. Almost all participants reported access to a latrine (*n* = 932, 98.6%), while 13 (1.4%) reported having none. Among households with sanitation facilities, water-sealed pit latrines were most common (*n* = 886, 95.1%), followed by ventilated pit latrines (*n* = 46, 4.9%).

### 3.2. Knowledge, Attitudes, and Practices Regarding HINIs

#### 3.2.1. Knowledge of the Participants

Overall, 61.7% (*n* = 583) demonstrated good knowledge, while 38.3% (*n* = 362) had poor knowledge. Most participants (*n* = 861, 91.1%) reported awareness of HINIs. Species-specific awareness (recognition based on commonly used names) varied by parasites. Pinworm (*E. vermicularis*; *n* = 859, 90.9%), hookworms (*N. americanus*/*Ancylostoma* spp.; *n* = 739, 78.2%), and large roundworm (*A. lumbricoides*; *n* = 529, 56.0%) were the most recognised. In contrast, whipworm (*T. trichiura*) was known by only 25.5% (*n* = 241), and no participants reported awareness of threadworm (*S. stercoralis*).

Knowledge of major transmission routes, such as faecally contaminated hands (*n* = 805, 85.4%) and contaminated food or water (*n* = 743, 78.7%), was relatively high. However, misconceptions were common, with mosquito bites (*n* = 250, 26.5%) and consumption of sweet foods (*n* = 755, 79.9%) identified as risk factors. Commonly recognised symptoms included abdominal pain (*n* = 849, 89.8%), loss of appetite (*n* = 795, 84.1%), and itching at entry sites (*n* = 756, 80.0%), whereas awareness of atypical manifestations, such as respiratory symptoms (*n* = 160, 16.9%), was limited. Complications like vitamin deficiencies (*n* = 569, 60.2%), malnutrition (*n* = 562, 59.5%), and anaemia (*n* = 502, 53.1%) were moderately recognised, while intestinal obstruction (*n* = 456, 48.3%) was less frequently identified (Table 1). Half of the participants recognised that infections may be asymptomatic (*n* = 474, 50.2%). Awareness of seasonal variation was limited, with 29.8% (*n* = 282) believing infections vary by season, and only 23.1% (*n* = 218) identifying the wet season as a higher risk period.

#### 3.2.2. Attitudes of the Participants

Most participants (*n* = 902, 95.6%) demonstrated positive attitudes towards HINIs (which is the perception that HINIs are harmful, preventable, and treatable, accompanied by a willingness to adopt preventive measures and seek appropriate treatment), while 4.6% (*n* = 43) showed poor attitudes. The majority recognised the importance of hygiene in preventing infection, with 92.5% (*n* = 874) agreeing that poor hygiene contributes to transmission and 90.7% (*n* = 857) believing that handwashing with soap reduces risk. Positive attitudes towards food hygiene were also common, with 90.6% (*n* = 856) identifying raw, unwashed fruits and vegetables consumption as a risk factor and 82.3% (*n* = 778) recognising risks associated with street food consumption. Preventive measures widely endorsed included keeping food and water covered (*n* = 805, 85.2%), trimming fingernails (*n* = 816, 86.3%), wearing footwear or gloves when handling soil (*n* = 723, 76.5%), and consistent latrine use (*n* = 820, 86.8%). Playing in sand was perceived as a risk by 77.5% (*n* = 732), and 72.4% (*n* = 684) believed regular bathing reduces transmission. Most participants believed deworming with an anthelmintic treatment prevents infection (*n* = 762, 80.6%) and is effective for treatment (*n* = 789, 83.5%). Opinions on traditional treatments were divided, with 36.2% (*n* = 342) supporting them and 38.5% (*n* = 364) disagreeing. A large majority recognised the potential health consequences of HINIs in children (*n* = 740, 78.3%), and 95.1% (*n* = 899) agreed that health education is a key strategy to reduce disease spread (Table 2).

#### 3.2.3. Practices of Caregivers

Overall, 99.2% (*n* = 937) of caregivers demonstrated good practices regarding HINIs. Self-reported handwashing practices were generally good. The most commonly reported practices were washing hands before feeding children (*n* = 919, 97.2%) and after handling diapers or faeces (*n* = 916, 96.9%). Washing of eating utensils before use was also commonly reported (*n* = 790, 83.6%). However, handwashing with soap before eating (*n* = 675, 71.4%) and before food preparation (*n* = 662, 70.0%) was less consistent. After latrine use, 74.2% (*n* = 701) reported washing hands with soap, 16.1% (*n* = 152) used water only, and 9.7% (*n* = 91) reported inconsistent practices (Table 3).

Food hygiene practices were generally good. Most caregivers reported washing raw foods (*n* = 845, 89.4%), consuming treated drinking water (*n* = 846, 89.5%), and keeping water and prepared food well-covered (*n* = 922, 97.6%). Personal hygiene practices were also favourable, with 82.4% (*n* = 779) reporting regular fingernail trimming, although 24.4% *(n* = 231) reported nail biting behaviour. Sanitation practices were strong overall, with 96.4% (*n* = 911) reporting consistent latrine use, though 3.6% (*n* = 34) reported occasional open defecation. Regular outdoor footwear use was reported by 60.4% (*n* = 571), while 26.3% (*n* = 249) wore footwear occasionally, and 13.2% (*n* = 125) reported not wearing footwear (Table 4).

#### 3.2.4. Practices of Children

Handwashing practices of the participating children, as reported by the caregivers, varied by context. Although 91.8% (*n* = 868) of children washed their hands with soap and water after defecation, only 55.7% (*n* = 526) did so before eating (Table 3). A majority (*n* = 667, 71.6%) washed raw foods before consumption, while 89.5% (*n* = 846) consumed treated drinking water. Sharing food at school was common, with 40.6% (*n* = 384) doing so regularly and 34% (*n* = 321) occasionally. Regular nail trimming was reported in 79.4% (*n* = 750), but nail-biting was highly prevalent (*n* = 783, 82.8%). Although most children used latrines consistently (*n* = 864, 91.4%), regular footwear use was low (*n* = 353, 37.2%), with 29.6% (*n* = 253) not using footwear at all. Frequent sandplay was seen among 70.7% (*n* = 667), and sharing of clothes and undergarments was uncommon (*n* = 823, 87.1% reported not sharing). Most households (n= 886, 93.8%) shared latrines within the family, while 1.3% (*n* = 13) without household latrines relied on neighbouring facilities (Table 4).

#### 3.2.5. Deworming Practices

More than half of caregivers (*n* = 559, 59.1%) reported continuing to administer anthelmintic treatment to their children despite the cessation of school-based deworming programmes. Among these caregivers, 51.5% (*n* = 487) administered treatment every three months, 18.8% (*n* = 178) every six months, and 1.3% (*n* = 12) annually, while 28.4% (*n* = 268) reported treatment only when symptoms occurred. Overall, 73.7% (*n* = 696) of children had received deworming medication within the year preceding the survey. Among these, 6.0% (*n* = 57) had received treatment within the previous two weeks, 51.2% (*n* = 484) between two weeks and one month, 38.2% (*n* = 361) between one and six months, and 4.6% (*n* = 43) between six and twelve months.

Most caregivers reported administering a single-dose regimen (*n* = 912, 96.5%), while 2.8% (*n* = 26) used multi-dose regimens (e.g., twice daily for three days), and 3.2% (*n* = 30) repeated a single dose after two weeks.

Deworming medication was most commonly obtained over the counter from pharmacies and self-administered (*n* = 676, 71.5%). A further 20.3% (*n* = 192) received treatment through hospital outpatient departments, while 8.1% (*n* = 77) obtained treatment from private general practitioners or clinics. Common reasons for self-administration included adherence to a self-defined regular deworming schedule and the perceived symptoms, such as hypopigmented facial patches, loss of appetite in the child, and perianal itching.

### 3.3. Association Between Sociodemographic Factors, Knowledge, Attitude, and Practices

**Figure 2:**
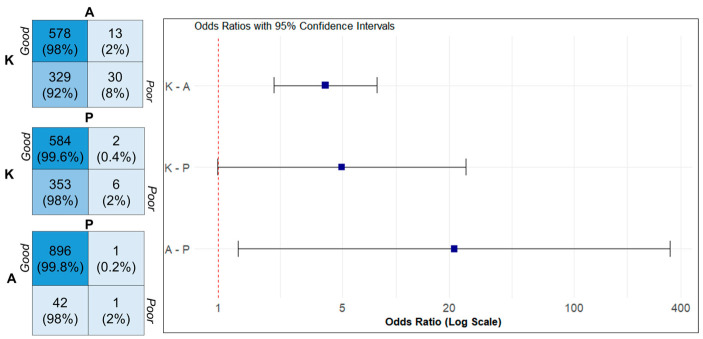
Heat map and forest plot depicting the associations between caregiver knowledge, attitudes, and practices, with odds ratios and 95% confidence intervals indicating the strength of relationships within the knowledge, attitude, and practice domains (*n* = 945). A, Attitudes; K, Knowledge; P, Practices.

A significant association was observed between knowledge and attitudes (χ^2^ = 19.16, df = 1, *p* < 0.001). Participants with good knowledge had higher odds of positive attitudes (OR = 4.02; 95% CI: 2.07–7.81), although the effect size was small (φ = 0.14). Knowledge was also significantly associated with practices (χ^2^ = 4.69, df = 1, *p* = 0.03). Participants having good knowledge showed higher odds of good practices (OR = 4.97; 95% CI: 1.0–24.71). No significant association was observed between attitudes and practices (χ^2^ = 1.17, df = 1, *p* = 0.28) (Figure 2).

There was no statistically significant association between caregivers’ knowledge of HINIs and participant educational level (primary, secondary and tertiary) (χ^2^ = 6.23, df = 5, *p* = 0.284), employment status (χ^2^ = 0.234, df = 1, *p* = 0.628), presence of a latrine (χ^2^ = 0.815, df = 1, *p* = 0.367) and household water treatment (χ^2^ = 0.893, df = 1, *p* = 0.345).

### 3.4. Association Between Caregiver KAP and HINIs in Children

**Figure 3:**
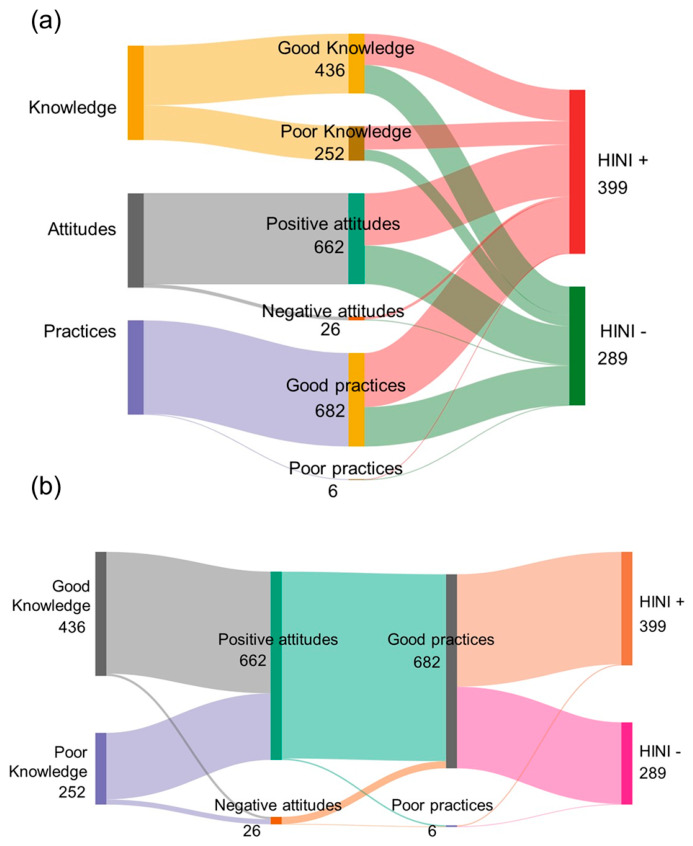
Alluvial diagrams showing associations between knowledge, attitude, practices and human intestinal nematode infection status. (**a**) Parallel alluvial diagram showing the distribution of participants across dichotomised knowledge (good/poor), attitude (positive/negative), and practices (good/poor) categories in relation to infection status (HINI positive/negative). Flows represent the number of individuals transitioning from each KAP domain to infection outcomes. (**b**) Sequential alluvial diagram illustrating the pathway from knowledge to attitude to practices and subsequently to HINI status. Node labels indicate the number and percentage of participants in each category. Flow widths are proportional to the number of individuals transitioning between categories. Detailed transition values are not shown to preserve visual clarity and are given in Supplementary Table S1. (+), Positive; (−), Negative.

Samples were obtained from 688 children, of whom 399 were positive for at least one HINI (*A. lumbricoides*, *T. trichiura*, hookworms, *S. stercoralis*, and *E. vermicularis*) (Figure 3). Diagnosis was performed using microscopy (direct saline and iodine smears, Kato–Katz, formalin–ether concentration, and agar plate culture methods) and quantitative PCR (qPCR). Associations between KAP variables and HINI were assessed among the 688 participants who provided stool samples. Detailed findings of the prevalence assessment have been reported elsewhere [20]. The relationships between KAP and HINI status are illustrated in an alluvial diagram (Figure 3), showing transitions across KAP domains and their association with infection outcomes. Sequential pathway analysis (Supplementary Table S1) demonstrated variation in HINI negativity across KAP combinations, with the highest negativity observed among individuals with good knowledge, attitude, and practice (48.5%); however, no consistent monotonic gradient was observed across the KAP sequence.

A significant association was observed between caregiver knowledge and presence of HINIs in children (χ^2^ = 15.88, df = 1, *p* < 0.001). Higher caregiver knowledge was associated with significantly lower odds of infection (OR = 0.519, 95% CI: 0.357–0.718). No significant association was observed between caregiver attitude (χ^2^ = 2.52, df = 1, *p* = 0.11) or practices (χ^2^ = 0.19, df = 1, *p* = 0.67) and child infection status (Table 5).

However, deworming practices were significantly associated with infection status (χ^2^ = 8.92, df = 1, *p* = 0.003). Children who had received deworming had lower odds of HINIs (OR = 0.58; 95% CI: 0.40–0.83), corresponding to a 42% reduction in odds, although the effect size was small (φ = 0.11).

In the univariate analysis, several knowledge, attitude, practice (KAP), and environmental factors showed significant associations with HINI status. Children with good knowledge had significantly lower odds of infection compared to those with poor knowledge (OR = 0.52, 95% CI: 0.37–0.72, *p* < 0.001). Regular deworming was also associated with reduced odds of HINI (OR = 0.58, 95% CI: 0.40–0.83, *p* = 0.003). Among environmental factors, use of treated drinking water (OR = 0.63, 95% CI: 0.43–0.92, *p* = 0.018), reported latrine usage (OR = 0.35, 95% CI: 0.19–0.66, *p* < 0.001), appropriate handwashing practices (OR = 0.64, 95% CI: 0.43–0.95, *p* = 0.028), absence of nail biting (OR = 0.55, 95% CI: 0.38–0.80, *p* = 0.002), and footwear usage (OR = 0.69, 95% CI: 0.51–0.94, *p* = 0.022) were all significantly associated with lower odds of infection. Attitudes and overall practices were not significantly associated with HINI but showed compatible odds with caregivers with positive attitudes and good practices having lower odds of developing HINIs. Caregiver status showed no significant association (*p* = 0.601), and sand play was also not significantly associated with infection status (*p* = 0.187). Overall, these findings indicate that both knowledge-related and selected hygiene-related behaviours are important protective factors in the unadjusted analysis of HINIs (Table 5).

Multivariable binary logistic regression analysis identified factors associated with HINI status (Table 6). The main effects model demonstrated a significant improvement in fit over the null model (Δχ^2^ = 59.53, *p* < 0.001; Nagelkerke R^2^ = 0.111). Inclusion of a knowledge × deworming interaction term further improved model fit (Δχ^2^ = 5.41, *p* = 0.02; Nagelkerke R^2^ = 0.121), and this model was retained as the final model. Several other tested interaction terms did not significantly improve model fit and were therefore not retained. In the final model, higher knowledge (OR = 0.41, 95% CI: 0.28–0.61, *p* < 0.001) and deworming (OR = 0.44, 95% CI: 0.28–0.71, *p* < 0.001) were independently associated with reduced odds of HINI. A statistically significant interaction between knowledge and deworming was observed (OR = 2.47, 95% CI: 1.15–5.28, *p* = 0.020), indicating effect modification, whereby the association between deworming and HINI differed according to caregiver knowledge level. Latrine usage (OR = 0.36, *p* = 0.002) and absence of nail biting (OR = 0.54, *p* = 0.002) were also significantly associated with lower odds of HINI, while other variables were not statistically significant.

## 4. Discussion

This study demonstrated that caregivers exhibited moderate knowledge, generally favourable attitudes, and good hygienic practices regarding HINIs, although important misconceptions and behavioural gaps regarding transmission routes, risk factors, and parasite species persisted. Hygiene-related practices among caregivers were generally favourable, yet several behaviours among children, including inconsistent footwear use and nail-biting, increase exposure risk. Importantly, higher caregiver knowledge was associated with lower odds of HINIs in children, whereas attitudes and practices were not independently associated with infection status. Regular deworming was also associated with reduced odds of infection, highlighting the continued importance of preventive chemotherapy even in settings where routine school-based deworming programmes have been discontinued. The KAP pathway analysis revealed that improvements in KAP do not translate linearly into reduced infection risk, as infections persisted even among households with good KAP scores. This indicates that behavioural measures alone may not fully capture exposure risk, likely reflecting ongoing environmental contamination, unmeasured behavioural variability, or residual confounding. These findings highlight the need to complement behaviour-focused interventions with environmental and structural control measures to effectively reduce transmission.

In post-deworming settings, such as the present context, where school-based deworming was discontinued in 2019 following previously low prevalence estimates [16], sustained surveillance becomes critical not only for infection monitoring but also for behavioural risk assessment. Low awareness of persistent transmission pathways may facilitate silent maintenance of infection, particularly in asymptomatic cases. Evaluating KAP, therefore, provides essential contextual evidence to identify behavioural vulnerabilities and inform targeted community interventions that support sustained control and prevention efforts.

To our knowledge, this represents the first assessment of KAP related to HINIs in the Anuradhapura district and in a post-deworming setting in Sri Lanka. The findings, therefore, provide timely behavioural evidence in the context of programme withdrawal. While the present analysis focuses on behavioural determinants (KAP), the detailed parasitological epidemiology and diagnostic evaluation conducted in the same population are reported separately. Our results showed that more than one-third of caregivers (38.2%) had poor knowledge, despite very favourable attitudes and generally good preventive practices. Similar knowledge gaps have been reported across several endemic and post-control settings in Asia and Africa [5,29,30], suggesting that deficiencies in community awareness remain common even where control programmes have substantially reduced infection prevalence. In contrast, positive attitudes and reported hygiene behaviours appear relatively consistent across many KAP studies on HINIs [6,7]. However, favourable attitudes do not always translate into sustained preventive practices, particularly where environmental, cultural, or behavioural factors influence daily activities [30]. This gap between awareness and behaviour is increasingly recognised as an important challenge for sustaining gains achieved through deworming programmes.

Despite overall positive findings, several knowledge gaps identified may increase vulnerability to HINIs. Awareness about *Ascaris*, *Trichuris*, and *Strongyloides* was low, and few participants recognised skin penetration as a transmission route. Knowledge on clinical manifestations and complications was also limited, while misconceptions such as attributing infection to sweet foods or polluted air were common. These patterns may partly reflect the low-prevalence context within the district [16], where exposure to symptomatic infections is uncommon. In contrast, studies from high-endemic settings often report poorer overall knowledge but greater recognition of symptoms and transmission routes due to frequent exposure to clinical disease [7]. Limited awareness of symptoms and complications can delay healthcare-seeking and facilitate continued household transmission, particularly where infections remain subclinical or mild [31].

Educational level, occupation, and household economic status were not significantly associated with knowledge of HINIs in this population, contrasting with many global studies, where education is a key determinant [7]. This may reflect the strong influence of community-based health education, particularly through public health midwives, who serve as a primary source of information on HINIs via routine home visits and maternal and child health clinics. Given that most participants were mothers of PSC, their knowledge is likely to have been acquired predominantly through these community health services rather than formal education [30,32]. However, it is also possible that the absence of an association reflects a more generalised dissemination of basic health information in this setting, where health messaging reaches households across educational strata, thereby reducing inequities in knowledge. In contrast, in settings where knowledge correlates with education, information is predominantly delivered through school-based health education or digital platforms, thereby favouring individuals with higher educational attainment [7]. Overall, these findings highlight the potential role of community health platforms in promoting equitable knowledge dissemination, while also demonstrating the need to further investigate how different information pathways interact with socioeconomic determinants [11].

The significant interaction between caregiver knowledge and deworming observed in the multivariable model suggests potential effect modification, in which the association between deworming practice and HINI infection varies according to the caregiver’s level of knowledge. This may indicate that the protective effect of regular deworming is more pronounced among children whose caregivers have higher knowledge, potentially due to better adherence to deworming schedules and reinforcement of preventive behaviours. Conversely, lower caregiver knowledge may limit the sustained effectiveness of deworming, possibly due to reduced compliance or continued exposure risk. Together, these findings support the importance of integrated, context-specific control strategies that combine deworming programmes with targeted health education interventions. However, causal interpretation is limited by the cross-sectional design, and the observed interaction should be interpreted as hypothesis-generating.

Attitudes towards prevention were highly favourable, with strong recognition of the importance of hygiene, safe food handling, and sanitation practices. High national coverage of WASH infrastructure may partly explain these positive perceptions. According to the WHO/UNICEF Joint Monitoring Programme (JMP) estimates, approximately 92% of the population uses improved drinking water sources, 94% uses improved sanitation facilities, and around 88% of households have access to handwashing facilities with soap and water [11,33]. These conditions likely support better household hygiene practices and reduced environmental contamination, thereby limiting the transmission of HINIs. However, infrastructure improvements alone are insufficient to interrupt transmission without sustained behavioural adherence to hygienic practices [34].

Although sanitary practices were generally satisfactory, several concerning behaviours were identified. The persistence of open defaecation, limited footwear use, and unhygienic behaviours such as nail-biting may provide opportunities for soil contamination and increase the risk of infection, particularly among children. Frequent soil contact and inadequate hygiene further heighten children’s vulnerability, while similar behaviours among adults may sustain environmental contamination and facilitate household transmission, even in low-prevalence settings.

The interpretation of the practice-related findings should take into account the highly skewed distribution of practice scores. Nearly all participants were classified as having good practices, leaving only a very small number in the poor-practice category. This limited variability may have produced a ceiling effect, reducing the statistical power to detect meaningful differences in infection status between groups. Therefore, the absence of a statistically significant association for the composite practice score should be interpreted with caution.

These findings have important implications for control strategies in low-prevalence and post-control settings. As countries reduce the frequency of MDA following successful reductions in infection prevalence, behavioural and environmental determinants of transmission become increasingly important. Targeted health education, school-based hygiene promotion, and sustained community engagement are therefore essential to reinforce preventive behaviours and prevent re-establishment of transmission. Integrating behavioural surveillance tools, such as periodic KAP assessments, into routine monitoring frameworks could provide early warning indicators of behavioural risk and guide targeted interventions that sustain the long-term impact of control programmes. Such behavioural data can complement parasitological surveillance by identifying transmission vulnerabilities before measurable increases in infection prevalence occur.

This study has several limitations. Data were collected using an interview-administered questionnaire, which may have introduced interviewer and social desirability bias. The exclusive focus on caregivers of PSC, most of whom were young females, presents both a limitation and a strength of this study. While perspectives from other demographic groups were not captured, caregivers represent a key population influencing household hygiene practices, sanitation behaviours, treatment adherence, and participation in deworming programmes [35]. Children are also particularly vulnerable to the nutritional and developmental consequences of HINIs, and may act as reservoirs sustaining community transmission. Understanding caregiver KAP, therefore, is vital and provides essential insights into behavioural and structural determinants of infection risk at the household level. However, findings may not be generalisable beyond the study setting, and residual confounding from unmeasured factors cannot be excluded. The cross-sectional design limits the ability to infer causal relationships between KAP factors and HINI infection.

Taken together, these findings indicate that maintaining progress in HINI control requires not only continued epidemiological surveillance but also attention to community knowledge and behaviour, an often overlooked yet critical component of long-term transmission interruption strategies.

## 5. Conclusions

Caregiver knowledge is an important behavioural determinant influencing preventive practices and infection outcomes related to HINIs. Higher caregiver knowledge was associated with significantly lower odds of infection among children. Although attitudes and reported hygiene practices were generally favourable, persistent misconceptions and specific behavioural risks, particularly among children, may continue to facilitate low-level transmission. These findings highlight that sustaining gains in HINIs control requires continued behavioural reinforcement alongside epidemiological surveillance. Even in low-prevalence settings where routine deworming programmes have been scaled down, targeted health education, school-based hygiene promotion, and community engagement remain essential to prevent resurgence and support the long-term sustainability of control efforts.

## Figures and Tables

**Table 1:** Knowledge of human intestinal nematode infections among participants (*n* = 945).

Knowledge Item	Categories	Response [n (%)]	
		Yes	No
Types of HINIs	*Strongyloides stercoralis* (threadworm)	0 (0.0)	945 (100)
	*Trichuris trichiura* (whipworm)	241 (25.5)	704 (74.5)
	*Ascaris lumbricoides* (large round worm)	529 (56.0)	416 (44.0)
	*Necator americanus* and *Ancylostoma* spp. (hookworm)	739 (78.2)	206 (21.8)
	*Enterobius vermicularis* (pinworm)	859 (90.9)	86 (9.1)
Modes of transmission	By piercing the bare skin	338 (35.8)	607 (64.2)
	By faecally contaminated food and water	743 (78.7)	202 (21.4)
	Consumption of sweet foods	755 (79.9)	190 (20.1)
	Consumption of foods contaminated by flies	795 (84.1)	150 (15.9)
	By faecally contaminated hands	805 (85.2)	140 (14.8)
Risk factors for transmission	Mosquito bites	250 (26.5)	695 (73.5)
	Breathing polluted air	370 (39.2)	575 (60.8)
	Usage of human faeces as fertiliser	511 (54.1)	434 (45.9)
	Use unclean water for cooking/drinking	635 (67.2)	310 (32.8)
	Walking barefoot	684 (72.4)	261 (27.6)
	Playing on the soil	705 (74.6)	240 (25.4)
	Open defaecation	731 (77.4)	214 (22.6)
	Biting of nails	738 (78.1)	207 (21.9)
Symptoms of HINI	Respiratory symptoms	160 (16.9)	785 (83.1)
	Blood and mucous diarrhoea	231 (24.4)	714 (75.6)
	Watery diarrhoea	321 (34.0)	624 (66.0)
	Loss of weight	627 (66.4)	318 (33.6)
	Bloating	658 (69.6)	287 (30.4)
	Itching in the entry site	756 (80.0)	189 (20.0)
	Loss of appetite	795 (84.1)	150 (15.9)
	Abdominal pain	849 (89.8)	96 (10.2)
Complications of HINIs	Intestinal obstruction	456 (48.3)	489 (51.7)
	Anaemia	502 (53.1)	443 (46.9)
	Malnutrition	562 (59.5)	383 (40.5)
	Vitamin deficiencies	569 (60.2)	376 (39.8)

**Table 2:** Attitudes of the participants towards the prevention of human intestinal nematode infections (*n* = 945).

Statement	Response [n (%)]			
I Think That	Agree	Neutral	Disagree	No Idea
using animal faeces as fertiliser increases the risk of HINIs	537 (56.8%)	87 (9.2%)	203 (21.5%)	117 (12.4%)
using human faeces as fertiliser increases the risk of HINIs	619 (65.5%)	78 (8.3%)	85 (9.0%)	161 (17.0%)
playing in the sand can increase the risk of HINIs	732 (77.5%)	63 (6.7%)	100 (10.6%)	49 (5.2%)
sharing clothes and toys can increase the transmission of HINIs	747 (79.0%)	58 (6.1%)	101 (10.7%)	39 (4.1%)
food prepared outdoors or eating street foods increases the risk of HINIs	778 (82.3%)	42 (4.4%)	80 (8.5%)	45 (4.8%)
raw, unwashed fruits and vegetables consumption can cause HINIs	856 (90.6%)	22 (2.3%)	49 (5.2%)	18 (1.9%)
lack of hygiene causes HINIs	874 (92.5%)	19 (2.0%)	35 (3.7%)	16 (1.7%)
washing hands only with water prevents HINIs	117 (12.4%)	43 (4.6%)	762 (80.6%)	23 (2.4%)
regular bathing reduces the risk of transmission of HINIs	684 (72.4%)	66 (7.0%)	155 (16.4%)	39 (4.1%)
the usage of treated water can prevent HINIs	707 (74.8%)	86 (9.1%)	106 (11.2%)	46 (4.9%)
wearing footwear and gloves when handling soil prevents HINIs	723 (76.5%)	77 (8.1%)	99 (10.5%)	46 (4.9%)
keeping food and water covered can prevent HINIs	805 (85.2%)	40 (4.2%)	81 (8.6%)	19 (2.0%)
keeping fingernails trimmed can prevent HINIs	816 (86.3%)	40 (4.2%)	64 (6.8%)	25 (2.6%)
latrine usage can prevent HINIs	820 (86.8%)	27 (2.9%)	70 (7.4%)	27 (2.9%)
household and surrounding cleanliness can reduce the prevalence of HINIs	841 (89.0%)	30 (3.2%)	52 (5.5%)	21 (2.2%)
washing hands with soap and water can prevent HINIs	857 (90.7%)	35 (3.7%)	38 (4.0%)	15 (1.6%)
traditional treatment is more effective for deworming than Western treatment	342 (36.2%)	153 (16.2%)	364 (38.5%)	86 (9.1%)
HINIs can be prevented using deworming	762 (80.6%)	78 (8.3%)	89 (9.4%)	16 (1.7%)
HINIs can be treated by deworming	789 (83.5%)	89 (9.4%)	51 (5.4%)	16 (1.7%)
HINIs can cause health complications, especially in children	740 (78.3%)	51 (5.4%)	63 (6.7%)	90 (9.5%)
health education can reduce the prevalence of HINIs	899 (95.1%)	18 (1.9%)	11 (1.2%)	17 (1.8%)

**Table 3:** Frequency distribution of handwashing practices among participants (*n* = 945).

Activity		Response [n (%)]				
		Most of the Time with Soap and Water	Most of the Time with Water Only	Sometimes with Soap and Water	Sometimes with Water Only	Never
Before eating	Caregiver	675 (71.4)	151 (16.0)	101 (10.7)	11 (1.2)	7 (0.7)
	Child	526 (55.7)	258 (27.3)	148 (15.7)	13 (1.4)	0 (0.0)
After latrine usage	Caregiver	701 (74.2)	152 (16.1)	53 (5.6)	38 (4.0)	1 (0.1)
	Child	868 (91.8)	43 (4.6)	24 (2.5)	10 (1.1)	0 (0.0)
Before feeding *		919 (97.2)	17 (1.8)	8 (0.8)	1 (0.1)	0 (0.0)
Before food preparation *		662 (70.0)	207 (21.9)	64 (6.8)	12 (1.3)	0 (0.0)
After handling baby diapers/excreta *		916 (96.9)	26 (2.7)	3 (0.3)	0 (0.0)	0 (0.0)
Washing eating utensils before use *		790 (83.6)	137 (14.5)	11 (1.2)	7 (0.7)	0 (0.0)

* Caregiver practices.

**Table 4:** Frequency distribution of sanitary practices (*n* = 945).

Practice	Response [n (%)]					
	Caregiver			Child		
	Mostly	Sometimes	No	Mostly	Sometimes	No
Washing things eaten raw	845 (89.4)	86 (9.1)	14 (1.5)	677 (71.6)	237 (25.1)	31 (3.3)
Use treated water for drinking	846 (89.5)	45 (4.8)	54 (5.7)	846 (89.5)	44 (4.7)	55 (5.8)
Keeping food/water covered	922 (97.6)	13 (1.4)	10 (1.1)	384 (40.6)	321 (34.0)	240 (25.4)
Trim fingernails regularly	779 (82.4)	99 (10.5)	67 (7.1)	750 (79.4)	130 (13.8)	65 (6.9)
Has a habit of biting nails	142 (15.0)	89 (9.4)	714 (75.6)	313 (33.1)	470 (49.7)	162 (17.1)
Latrine usage for defecation	911 (96.4)	31 (3.3)	3 (0.3)	864 (91.4)	42 (4.4)	39 (4.1)
Regular footwear usage outdoors	571 (60.4)	249 (26.3)	125 (13.2)	352 (37.2)	313 (33.1)	280 (29.6)
The child’s habit of playing in the sand				668 (70.7)	167 (17.7)	110 (11.6)
The child’s habit of sharing food				384 (40.6)	321 (34)	240 (25.4)
Sharing of clothes and undergarments				0 (0)	122 (12.9)	823 (87.1)

**Table 5:** Associations between caregiver knowledge, attitude, practices, sanitary facilities, deworming behaviour, and child human intestinal nematode infection status (*n* = 688).

Variable	Category	HINI +	HINI −	χ^2^ (df = 1)	OR (95% CI)	*p*-Value
Knowledge	Good	228	208	15.88	0.52 (0.37–0.72)	<0.001
	Poor	171	81			
Attitudes	Positive	380	282	2.52	0.49 (0.20–1.20)	0.112
	Negative	19	7			
Practices *	Good	395	287	0.19	0.69 (0.12–3.78)	1.00 *
	Poor	4	2			
Deworming	Regular	277	230	8.92	0.58 (0.40–0.83)	0.003
	Irregular/symptom-based	122	51			
Caregiver ^#^	Full-time caregiver	354	260	0.27	0.88 (0.54–1.44)	0.601
	Other caregiver	45	29			
Drinking water	Treated at home	65	68	5.63	0.63 (0.43–0.92)	0.018
	Not treated	334	221			
Latrine usage	Present	349	275	11.74	0.35 (0.19–0.66)	<0.001
	Absent	50	24			
Handwashing	Consistent	310	244	4.85	0.64 (0.43–0.95)	0.028
	Inconsistent	89	45			
Nail biting	Present	333	213	9.74	0.55 (0.38–0.80)	0.002
	Absent	66	76			
Footwear use	Present	143	129	5.43	0.69 (0.51–0.94)	0.022
	Absent	256	160			
Play in the sand	Present	347	261	1.82	0.70 (0.42–1.16)	0.177
	Absent	53	28			

OR, odds ratio; CI, confidence interval; HINIs, human intestinal nematode infections; χ^2^, chi-square. **^#^** All full-time caregivers were mothers. * Fisher’s exact *p*. *p*-values were calculated using the chi-square test. Fisher’s Exact test was used where expected cell counts were <5.

**Table 6:** Multivariable logistic regression analysis of factors associated with human intestinal nematode infection (*n* = 688).

Variable	Category	Adjusted Odds Ratio	95% Confidence Interval	*p*-Value
Knowledge	Good/Poor	0.41	0.28–0.61	<0.001
Deworming	Regular/Irregular	0.44	0.28–0.71	<0.001
Caregiver	Full-time/Part-time	0.81	0.48–1.37	0.437
Drinking water	Treated/Non-treated	0.67	0.45–1.01	0.056
Lareine usage	Present/Absent	0.36	0.19–0.68	0.002
Regular handwashing	Consistent/Inconsistent	0.68	0.45–1.03	0.067
Nail biting	Present/Absent	0.54	0.36–0.80	0.002
Sand play	Present/Absent	1.35	0.81–2.25	0.248
Footwear usage	Present/Absent	0.75	0.54–1.03	0.077
Knowledge × Deworming	Interaction	2.47	1.15–5.28	0.020

## Data Availability

All data generated or analysed during this study are included in this published article and its Supplementary Materials.

## Supplementary Materials

The following supporting information can be downloaded at: https://www.mdpi.com/article/10.3390/tropicalmed11070191/s1. Table S1: Sequential pathway from knowledge to attitude to practices and subsequently to HINI status.

## Abbreviations

The following abbreviations are used in this manuscript:

HINIs	human intestinal nematode infections
STH	Soil-transmitted helminths
WASH	water, sanitation, and hygiene
MDA	mass drug administration
KAP	knowledge, attitudes, and practices
WHO	World Health Organization
NWSDB	National Water Supply and Drainage Board
CKDu	chronic kidney disease of uncertain aetiology
RO	reverse osmosis
QGIS	Quantum Geographic Information System
CI	confidence interval
SPSS	Statistical Package for the Social Sciences
qPCR	quantitative PCR
OR	odds ratio
SD	standard deviation
