# Supplementary material for: Behavioural Determinants of Intestinal Nematode Infection Risk Among Children in a Post-Mass-Drug-Administration Setting in Sri Lanka: A Survey of Caregiver Knowledge, Attitudes, and Practices
Source: Trop Med Infect Dis. 2026 Jul 9;11(7):191. doi: 10.3390/tropicalmed11070191 (PMC13417374; doi:10.3390/tropicalmed11070191)
Supplement: Supplementary file 1 [file tropicalmed-11-00191-s001.zip › tropicalmed-4376086-supplementary.pdf]

Supplementary Table S1. Sequential pathway from knowledge to attitude to practices and subsequently to HINI status.

**Knowledge**	**Attitude**	**Practice**	**Total (n)**	**HINI + (n)**	**HINI – (n)**	**Positivity (%)**	**Negativity (%)**
Poor	Poor	Poor	1	0	1	0	**100**
Poor	Poor	Good	17	12	5	70.6	**29.4**
Poor	Good	Poor	4	3	1	75	**25**
Poor	Good	Good	230	156	74	67.8	**32.2**
Good	Poor	Poor	0	0	0	—	**—**
Good	Poor	Good	8	7	1	87.5	**12.5**
Good	Good	Poor	1	1	0	100	**0**
Good	Good	Good	427	220	207	51.5	**48.5**

*Percentages represent within-pathway proportions. This table corresponds to the complete set of KAP transitions illustrated in Figure 3(b).
